# Next-Generation Biomarkers in Multiple Myeloma: Advancing Diagnosis, Risk Stratification, and Precision Therapy Beyond Current Guidelines

**DOI:** 10.3390/ph19020320

**Published:** 2026-02-14

**Authors:** Marta Marques de Carvalho Lopes, Laura do Amaral Xavier, Silvia Cristina Verde Mendes Nolasco, Simone Rodrigues Ribeiro, Danila Felix Coutinho, Adriano de Paula Sabino

**Affiliations:** Postgraduate Program in Clinical and Toxicological Analysis, Department of Clinical and Toxicology Analysis, College of Pharmacy, Federal University of Minas Gerais, Prof. Moacir Gomes de Freitas Street-Pampulha, Belo Horizonte 31270-901, MG, Brazil; marta.mdecarvalho@gmail.com (M.M.d.C.L.);

**Keywords:** multiple myeloma, biomarkers, precision medicine, multiomics, targeted therapy

## Abstract

Multiple myeloma (MM) is an oncohematological neoplasm characterized by the abnormal proliferation of neoplastic plasma cells in the bone marrow and the excessive secretion of monoclonal antibodies into the bloodstream. Approximately 3 to 5% of patients present with a variant form of the disease where there is no secretion of monoclonal proteins, characterizing the non-secretory MM picture. It exhibits a highly complex and heterogeneous genetic signature, allowing the disease to be classified into premalignant entities and symptomatic forms. In this context, an integrative narrative review was conducted, encompassing genomic, epigenomic, proteomic, metabolomic, and radiomic biomarkers described in the literature between 2018 and 2025. Emphasis was placed on their translational potential, current limitations in clinical practice, and gaps within recent recommendations. Several categories of biomarkers, particularly ctDNA methylome, single-cell multiomics, proteomics of surface antigens, functional ex vivo assays, and PET/CT radiomics, demonstrate strong potential for enhancing risk stratification, detecting early progression, guiding therapy selection, and identifying novel therapeutic targets. These applications extend beyond existing guideline frameworks. Thus, integrating advanced biomarker platforms can overcome limitations of current diagnostic and therapeutic models and enhance precision strategies across plasma cell disorders.

## 1. Introduction

Multiple myeloma (MM) is the second most common type of hematological cancer, surpassed only by non-Hodgkin’s lymphomas; it is characterized by the abnormal proliferation of clonal neoplastic plasma cells in the bone marrow and the secretion of large amounts of monoclonal into the bloodstream. Clinically, the manifestations caused by this plasma cell dyscrasia are defined by the SLiM-CRAB criteria, which include hypercalcemia (C), renal dysfunction (R), anemia (A), and osteolytic lesions (B), and the presence of one myeloma-defining disorder. Approximately 3 to 5% of patients present with a variant form of the disease where there is no secretion of monoclonal proteins, characterizing the non-secretory MM picture [1,2].

**Figure 1:**
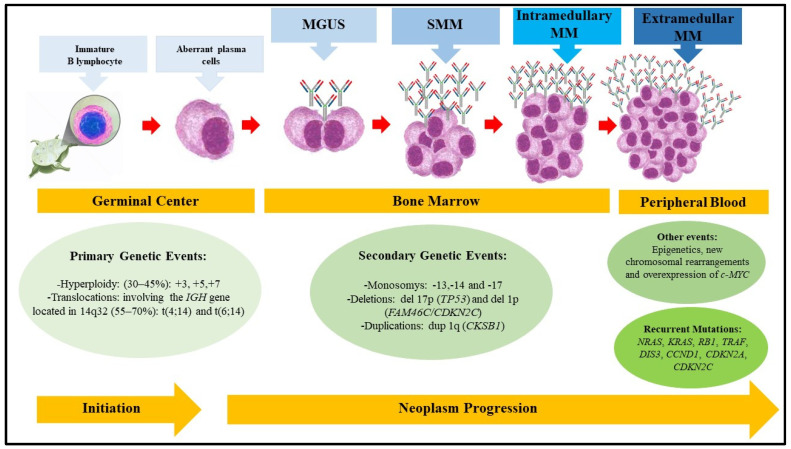
Progression of multiple myeloma. Schematic illustration of disease progression. Initially, an immature B lymphocyte undergoes mutational processes in the germinal center of secondary lymphoid organs. These mutations, known as primary genetic events, lead to the emergence of immortalized, aberrant clonal plasma cells. These cells migrate to the bone marrow and begin producing large quantities of monoclonal antibodies, resulting in the first premalignant entity of the disease, monoclonal gammopathy of uncertain significance (MGUS). Subsequently, additional secondary genetic events occur and accumulate, leading to smoldering multiple myeloma (SMM), the second premalignant entity. The resulting genomic instability favors the emergence of new chromosomal rearrangements and mutations in oncogenes and/or tumor suppressor genes, as well as epigenetic alterations, thereby promoting progression to the symptomatic form of the disease. Ultimately, a very rare form of the disease, extramedullary multiple myeloma, may develop.

The complex set of genetic, molecular, and epigenetic alterations involved in the course of the disease allows it to be divided into premalignant and symptomatic entities. Benign monoclonal gammopathy of uncertain significance (MGUS) and smoldering or indolent multiple myeloma (SMM) are classified as premalignant entities. In contrast, plasma cell leukemia and intramedullary or extramedullary symptomatic multiple myeloma are malignant, more aggressive, and widespread forms of the disease. These premalignant stages begin to emerge after primary genetic events, including chromosomal translocations in regions containing the immunoglobulin heavy chain *IGH* genes and aneuploidies. Additional secondary changes may subsequently occur, including DNA hypomethylation, copy number abnormalities, and acquired mutations, ultimately leading to disease progression (Figure 1) [1,3,4].

After the elucidation of tumor pathogenesis, which established that every patient with symptomatic multiple myeloma has, at some point in their lifetime, presented with MGUS and SMM, the detection of new, specific biomarkers in each phase of the disease, which might potentially be combined, seems highly promising and predictive, especially since only patients with early active and symptomatic myeloma are currently being treated. Progression from MGUS/SMM to MM is heterogeneous, and early intervention is not recommended outside clinical trials, except for individuals at high risk. A major clinical challenge is accurately identifying individuals at high risk of progression who might benefit from preventive or early-intervention strategies [5,6,7].

In this context, precision medicine has gained popularity, because recognizing the heterogeneity of the neoplasm and considering each patient’s genetic profile allows for highly promising, less invasive, and more targeted therapeutic approaches. Emerging technologies, such as liquid biopsy, multiomic profiling, and radiomics, offer opportunities to overcome limitations of bone marrow-restricted diagnostics by capturing spatial and temporal tumor heterogeneity. These tools may refine prognostication, enable earlier detection of progression, and support personalized treatment strategies [8,9]

Thus, the objective of this review was to provide a concise overview of foundational concepts in plasma cell disorders and to evaluate how emerging biomarker platforms may support new diagnostic and therapeutic precision-medicine strategies not yet incorporated into NCCN (National Comprehensive Cancer Network) or IMWG (International Myeloma Working Group) guidelines.

### 1.1. Premalignant Entities and Risk of Progression

The monoclonal gammopathy of uncertain significance (MGUS) is defined by the presence of serum MP < 3.0 g/dL, fewer than 10% clonal plasma cells (PCs) in the bone marrow, and no evidence of organ damage as established by the SLiM-CRAB criteria. Population screening studies have shown that the presence of paraproteins in peripheral blood can begin in individuals after the age of 30 and is observed in about 5% of the global population above the age of 50. These findings are generally detected incidentally during routine medical examinations or investigations of infectious symptoms, as there are still no established screening guidelines. The annual risk of progression to MM, as well as to other benign conditions such as amyloidosis and macroglobulinemia, is roughly 1% per year [10,11,12].

As previously noted, predicting which individuals with an initial diagnosis of MGUS will progress to multiple myeloma remains a substantial challenge. A comprehensive clinical evaluation and structured longitudinal monitoring are therefore essential to identify early signs of disease evolution and to exclude alternative malignant or amyloidogenic processes. Risk stratification is commonly based on the Mayo Clinic model, which incorporates the serum M-protein concentration, immunoglobulin isotype, serum free light-chain (FLC) ratio, and bone marrow plasma cell involvement. According to these parameters, MGUS can be categorized as low, intermediate, or high risk, while light-chain MGUS is assessed separately using FLC-based criteria. These classifications guide the intensity of follow-up and additional testing (Table 1). The IMWG recommends that low-risk MGUS be monitored at 6 months and, if stable, every 2–3 years thereafter, whereas intermediate- and high-risk MGUS should be followed every 6–12 months. Bone marrow biopsy is not routinely required for IgG MGUS unless red flags such as anemia, renal impairment, hypercalcemia, or unexplained bone pain are present [1,12,13,14,15].

Smoldering myeloma (SMM) is also an asymptomatic clinical condition, situated between MGUS and MM. For its diagnosis, an M protein (MP) level ≥ 3.0 g/dL, 10–60% neoplastic plasma cells in the bone marrow, and absence of SLiM-CRAB criteria are needed. From a clinical standpoint, distinguishing SMM from MGUS is extremely important, since upon initial detection, the risk of progression to cancer is approximately ten times higher. Its subsequent risk of progression, however, is more predictable and 10% in the first five years, and 3% in the following five years. The findings of Mateos et al. [16] corroborate and further reinforce that the first five years are indeed crucial, with a relatively higher risk [16]. Therefore, monitoring and counseling patients must be carried out in a more rigorous and targeted manner [1,16,17].

An understanding of the reduced risk of progression to active disease over time, together with laboratory findings, has allowed SMM to be clinically defined as a heterogeneous entity, rather than merely an intermediate stage between MGUS and MM, encompassing both indolent and progressive subgroups. Thus, individuals can be separated into those with biological premalignancy (indolent) and those with biological malignancy (progressive). In this framework, one of the initial goals was to identify which subgroup of SMM patients with biological malignancy would develop clinical complications within two years. These new approaches have driven major paradigm shifts over the past decade, providing novel treatment options. A new concept within the diagnostic criteria revised by the International Myeloma Working Group (IMWG), termed SLiM (S-Sixty), (Li-Light Chains-FLC) and (M-MRI Lesions), emerged and was adopted in 2014 [1,18].

SLiM were validated and defined by three biomarkers: (S) clonal bone marrow plasma cells ≥60%, (Li) involved/uninvolved serum free light-chain (FLC) ratio ≥ 100, provided that the absolute level of the involved light chain is ≥100 mg/L, and (M) more than one focal bone lesion ≥5 mm in diameter as visualized on MRI more predictable. The presence of these three markers indicates a high risk around 80% of progression within two years. Under these circumstances, the disease is classified as early active myeloma based on SLiM or ultrahigh-risk indolent myeloma (HR-SMM), and treatment is recommended [1,19].

Both the IMWG and the NCCN agree that only patients with HR- SMM defined either by the presence of a myeloma-defining event or by validated risk models (20/2/20, Mayo 2018, or IMWG scoring) should be considered for early therapeutic intervention, preferably within clinical trials. Standard-risk SMM should not be treated and must instead be monitored at intervals of 3–6 months [1,20,21].

### 1.2. Multiple Myeloma

Multiple myeloma accounts for approximately 1% of all cancers and 10% of all hematological malignancies, making it the second most common oncohematological disease and primarily affecting men in their 60s and 70s. In 2019, about 156,000 new cases were diagnosed worldwide, of which 54.3% were in men. The establishment and growth of tumors within the bone marrow, in conjunction with the host response and serum M protein levels, lead to the development of clinical features that define the symptomatic phase of the disease [1,22,23].

All symptomatic MM cases originate from MGUS and SMM, which involve primary and secondary genetic aberrations that arise over the course of tumor progression. This makes multiple myeloma an extremely complex disease, only partially understood and still incurable. Its marked genetic heterogeneity is linked to interactions between tumor cells and the bone marrow microenvironment, driving clonal evolution and/or intraclonal variations that can impede prolonged complete remission in some patients [23].

NCCN guidelines recommend a comprehensive baseline evaluation including: complete blood count; renal, calcium and LDH (low-dessity lipoprotein) levels; SPEP, UPEP and immunofixation; serum free light-chain assays; and a bone marrow aspirate/biopsy with cytogenetics and FISH (Fluorescence In Situ Hybridization) for high-risk abnormalities (t(4;14), t(14;16), t(14;20), del(17p), 1q gain). For imaging, whole-body low-dose CT (WBLDCT) is preferred, with MRI or PET-CT (Positron Emission Tomography) when available [20].

## 2. Diagnosis and Prognosis

In 2014, the International Myeloma Working Group (IMWG) revised the diagnostic criteria for MM and related diseases. To be diagnosed, the patient must present at least one SLiM, target organ lesions defined by the CRAB criteria, at least one osteolytic lesion, and an FLC ratio ≥ 100 mg/L. Osteolytic bone lesions are found in a large proportion of patients (approximately 80%) and differ from other tumor types that often induce bone overgrowth. These lesions are invasive and irreversible, leading to osteoporosis, pathological fractures, and severe pain, directly interfering with the patient’s quality of life and often preventing them from performing daily tasks without assistance [1,10].

In 2022, the International Classification Consensus (ICC) proposed some changes to the terminology and diagnostic criteria for mature lymphoid neoplasms. This new classification differs only slightly from that adopted by the 2014 IMWG and the World Health Organization (WHO) 2016 classification. This same 2022 consensus also proposed precisely dividing the disease into two exclusive cytogenetic groups, the first called MM with recurrent genetic alterations, namely, translocations involving the *IGH* and *NSD2* genes, *CCND* and *MAF* families, and odd chromosome hyperploidy. The second group includes all other genetic changes and rearrangements, without further specifications. MM can be further subdivided into four exclusive cytogenetic groups: favorable, standard risk, low risk, and high risk. The genetic profile is highly important for the diagnosis, prognosis, and clinical course of the neoplasm. The Durie Salmon Staging (DSS), the International Staging System (ISS), and the Revised International Staging System (R-ISS) are the most commonly used staging systems in clinical practice [24,25,26].

The diagnostic and prognostic evaluation follows the IMWG and NCCN recommendations, which integrate clinical parameters, tumor burden (SLiM-CRAB), cytogenetic abnormalities identified by FISH, and advanced imaging. The Revised ISS (R-ISS) remains the standard, whereas the NCCN also incorporates high-risk cytogenetic markers when selecting therapy [1,20,27,28].

## 3. Treatment

Despite therapeutic progress, MM remains challenging to treat, particularly in cases with high-risk cytogenetic features, extramedullary disease, or early relapse. Innovative therapies, including CAR-T cells (ide-cel, cilta-cel), bispecific antibodies (teclistamab, elranatamab, talquetamab, linvoseltamab), CELMoDs, and antibody–drug conjugates, are reshaping the therapeutic landscape. These targeted therapies support personalized treatment approaches informed by disease biology, minimal residual disease (MRD) status, and emerging biomarker-guided precision strategies (Table 2) [20,27,29,30,31,32,33].

According to the NCCN version 4.2026 guidelines, the preferred induction regimen for transplant-eligible patients is daratumumab, bortezomib, lenalidomide and dexamethasone (Dara-VRd), based on improved depth of response and progression-free survival. Other acceptable options include VRd and Isa-VRd. For transplant-ineligible patients, the NCCN recommends Dara-Rd as a category 1 preferred regimen, with VRd or Rd being alternatives depending on frailty and comorbidities. The NCCN recommends lenalidomide maintenance for standard-risk patients, while those with high-risk cytogenetics may benefit from bortezomib-based or combination bortezomib/lenalidomide maintenance [20].

Recent advances have reshaped the management of high-risk smoldering multiple myeloma (HR-SMM). Daratumumab has become the first therapy approved for HR-SMM following the results of the phase III AQUILA trial, which demonstrated that early intervention can meaningfully delay progression to symptomatic disease. In this study, daratumumab subcutaneous monotherapy reduced the risk of progression to active multiple myeloma or death by 51% compared with active surveillance, with 5-year progression-free survival rates of 63.1% versus 40.8%, respectively. Early treatment also conferred a survival advantage, with a 5-year overall survival of 93% in the daratumumab arm versus 86.9% in the observation arm. These findings establish a new paradigm in which therapeutic intervention is justified in carefully selected HR-SMM patients, supporting a shift from the traditional “watch-and-wait” strategy toward a risk-adapted, disease-interception approach [34].

## 4. Precision Medicine

MM develops through a multistep biological continuum beginning with MGUS and SMM, driven by accumulating genomic, epigenomic, and microenvironmental alterations. Subclonal heterogeneity and clonal evolution contribute to disease progression, treatment resistance, and spatial genetic diversity. Most patients are diagnosed only after myeloma-defining organ damage is established, underscoring the need for biomarkers capable of detecting early biological transformation [35,36,37].

Clinical outcomes vary widely due to underlying biological heterogeneity. Advanced biomarker platforms including genomic, epigenomic, proteomic, metabolomic, radiomic, and functional assays offer opportunities for improved risk stratification, early detection of progression, and biologically guided therapy selection, beyond what current NCCN/IMWG guidelines incorporate [38].

Precision medicine aims to tailor diagnostics and treatment to tumor biology using integrated multiomic and imaging data. Such approaches improve diagnostic accuracy, refine prognostication, minimize ineffective therapies, and support the development of targeted agents. This review emphasizes biomarker-driven strategies that can advance precision diagnostics and therapy beyond existing guideline frameworks [37,38].

Liquid biopsy, an approach that seeks to detect tumor particles or cells in body fluids through minimally invasive methods, has generated significant interest in precision medicine. Bone marrow samples remain the standard tissue biopsies for diagnosing, prognosticating, and monitoring MM, yet collecting such samples is quite invasive and extremely painful, causing substantial patient discomfort. Moreover, it may not represent the entire genetic heterogeneity of the cancer. A single bone marrow aspirate, even if collected at different time points, may not capture the precise location of diseased plasma cell colonization, and the sample volume may be insufficient for comprehensive screening. Obtaining more aliquots from multiple sites and at different stages (diagnosis, prognosis, and monitoring) would be ideal, but these procedures are profoundly invasive and unfeasible for most patients [8,9,35].

Hence, the assessment of circulating tumor cells, extracellular RNA, cell-free DNA, tumor-modified platelets, extracellular vesicles, multiomics analyses and genome optical mapping (OGM), in peripheral blood or other body fluids, represents highly reliable and minimally invasive alternatives that can directly reflect tumor biology. Notably, none of these emerging biomarkers—CTCs, circulating miRNAs, exosome-derived signatures, tumor-educated platelets, cfDNA quantification, or multiomic algorithms—are included in the current NCCN or IMWG guidelines for diagnosis or treatment. These modalities have potential to enable MRD assessment, identify resistance mutations, track clonal evolution, and guide therapy decisions Their incorporation into clinical practice may redefine risk stratification and treatment selection beyond conventional cytogenetics and SLiM-CRAB criteria [39,40,41,42,43].

### 4.1. Circulating Tumor Cells (CTCs)

Circulating tumor cells have been reported in various types of neoplasms and are detected early in carcinogenesis. They are substantial biomarkers that provide essential information about the tumor. In MM, as tumor cells spread, they may leave and return to the bone marrow, where they can be found in peripheral blood. Recently, a study showed that CTCs were detected in 59% of patients with MGUS and 100% of patients with SMM and active MM [44,45].

### 4.2. Extracellular RNA (RNAs)

MicroRNAs (miRNAs) are the most abundant extracellular RNAs found in circulation. They are small molecules of approximately 19 to 24 nucleotides, very stable, and non-protein-coding. Identified as potent post-transcriptional regulators of gene expression in animals and plants, they were discovered in the 1990s. Due to their short sequences and action that does not require complete complementarity, miRNAs can regulate numerous target mRNAs, leading to markedly different biological functions [41,46,47]. Al Masri et al. (2005) first reported evidence of miRNA involvement in the pathogenesis of MM; MM patients and cell lines showed lower expression of miR-125b, miR-133a, miR-1, miR-124a, miR-15, and miR-16 compared with normal plasma cells [48].

### 4.3. Extracellular Vesicles

Every cell in the human body secretes lipid-layered nanoparticles known as extracellular vesicles (EVs). They are typically categorized by diameter and exhibit various biological and even pathological functions. EVs carry molecules from the origin cell and can be readily isolated from peripheral blood, making them excellent biomarkers for liquid biopsy. Exosomes illustrate a key type of EV; these nanovesicles measuring ~30–150 nm, when secreted by neoplastic cells, foster tumor progression in several cancer types. The transfer of miRNAs between cells is one among the numerous roles these nanoparticles fulfill. Multiple studies have highlighted the significant relevance of analyzing miRNAs extracted from exosomes in MM progression, enabling researchers to discern which miRNAs are upregulated or downregulated during the progression from MGUS to overt disease [42,49,50].

### 4.4. Tumor-Educated Platelets

Platelets are cell fragments derived from megakaryocytes, primarily recognized for their central roles in hemostasis. A healthy individual’s blood contains approximately 150–400 × 10^9^/L platelets, implying high bioavailability for potential clinical use [51]. In addition to being readily obtained, isolation is relatively straightforward, rendering them appealing biomarkers. As non-nucleated cell fragments, platelet RNA transcripts, inherited from the megakaryocyte, are crucial for functional maintenance. Thus, platelet RNA can be efficiently extracted and analyzed to assess gene expression. Nilsson et al. (2011) [43] demonstrated that platelets from healthy individuals are capable of absorbing mutant RNAs from tumor cells in patients with glioma and prostate cancer, both in vitro and in vivo, offering a potential tool in personalized medicine. To date, no specific studies addressing modified platelets in MM have been published; however, several other reports have explored relationships between MM and platelets [43].

### 4.5. Multiomics Analysis

Advances in technologies such as next-generation sequencing (NGS) have contributed significantly to understanding the genomic heterogeneity observed in multiple myeloma (MM), directly influencing prognosis and response to treatment. These treatments can now be individualized, contextualizing the focus of precision medicine [52,53]. Targeted NGS panels are used in clinical practice to detect recurrent mutations in genes such as *KRAS*, *NRAS*, *TP53*, *BRAF*, *FAM46C*, and *DIS3*. Detection of these mutations provides prognostic information and can guide the selection of targeted therapies, such as the use of *BRAF* inhibitors in patients with the V600E mutation. Additionally, these panels serve as valuable tools for disease monitoring and identification of resistance mechanisms [52,54]. Although there are commercially available panels for genomic analysis of patients with myeloma, most of them are developed for application in myeloid neoplasms in general. Therefore, in the context of multiple myeloma, some studies have been carried out to develop customizable panels, such as the clinical trial GEM12, conducted in Spain and initiated in 2017. This study included 149 patients and used a custom capture-based NGS panel designed according to the main genomic alterations seen in multiple myeloma, including genes located in the 14q32 region. The panel evaluated translocations, indels, and SNVs, and demonstrated highly accurate results, such as the mutational status of *TP53* and its association with clinical outcomes, as well as clonal rearrangements of *IGH* [55]. A study conducted by Ren et al. (2024) [53] showed that genomic sequencing of 35 patients exhibited high sensitivity and specificity for detecting IG gene rearrangements associated with multiple myeloma. According to the authors, the study also demonstrated that RNA-seq was more efficient than DNA-seq for MRD assessment due to its greater sensitivity [53].

Whole-exome sequencing (WES), although still limited to clinical research settings, allows for global analysis of the genome’s coding exons and has been useful in identifying mutations not detected by conventional panels. This approach contributes to the discovery of new biomarkers and therapeutic targets, particularly in cases of clonal evolution or myelomas with atypical genetic profiles. Recent studies have also employed WES in patients with myeloma. It is important to emphasize that exome sequencing represents a complementary alternative, as it allows the detection of point mutations that may not be revealed by DNA-seq. Additionally, it supports the inference of hyperploidy and aids in risk stratification. However, it is not capable of detecting certain rearrangements, such as those involving IG loci [56,57,58].

Recent large-scale NGS studies have shown that non-coding and complex structural genomic alterations also play a significant role in disease progression, even at early stages such as MGUS and smoldering myeloma. Furthermore, single-cell technologies have revealed previously undetectable tumor subclones and new interactions with the microenvironment, impacting therapeutic resistance and prognosis [57].

The transcriptome refers to the complete set of RNA (RNA-seq) molecules expressed in a cell or tissue at a specific time, reflecting gene activity and providing insights into gene regulation and biological processes under normal or pathological conditions. Tumors’ transcriptomes are remarkably useful for the interrogation of cancer phenotypes. Transcriptomic analysis through RNA-seq has emerged as a powerful approach for characterizing the molecular heterogeneity of multiple myeloma, enhancing diagnostic accuracy, enabling the identification of prognostic and predictive biomarkers, and supporting molecular disease classification [59]. In recent years, the development of RNA-seq-based gene signatures in MM has become a major research focus, aiming to improve risk stratification and guide therapeutic decision-making. Transcriptomic scores derived from RNA-seq data have shown substantial potential for prognostic assessment and personalized treatment planning. Based on comprehensive gene expression profiles, these scores enable more precise patient categorization and outcome prediction. Their integration into prognostic models has improved individual risk assessment and advanced precision medicine strategies in MM. Notably, Yu et al. (2022) identified an eight-gene signature related to hypoxia and immunity (*CHRDL1*, *DDIT4*, *DNTT*, *FAM133A*, *MYB*, *PRR15*, *QTRT1* and *ZNF275*) that effectively stratifies patient risk [60].

Despite its strengths, RNA-seq has important limitations in the clinical context of MM. Cellular heterogeneity in bone marrow samples may hinder the accurate detection of tumor-specific signatures, especially in cases with low plasma cell infiltration. Traditional bulk RNA-seq also fails to capture single-cell variability, limiting the identification of clinically relevant subpopulations. Furthermore, the need for high-quality RNA, elevated costs, and analytical complexity restrict its routine application in clinical settings. Importantly, RNA-seq measures only transcriptional output and does not directly assess epigenetic alterations or protein function, both of which are central to MM biology and drug resistance mechanisms [59].

Metabolomics, by dynamically reflecting the functional state of cells through the analysis of metabolites, has revealed significant alterations in energy, lipid, and oxidative metabolic profiles in patients with multiple myeloma, particularly in advanced stages of the disease. Its application has shown promise in diagnosis, prognosis, and therapeutic monitoring [61]. Yue et al. (2022) identified altered amino acid profiles for choline, creatinine, leucine, tryptophan, and valine that correlated with clinical parameters, suggesting their potential as diagnostic biomarkers in multiple myeloma [61]. Liu et al. (2023) [62] demonstrated that Pim-2 kinase, which is frequently overexpressed in multiple myeloma, plays a key role in regulating cellular energy metabolism. Inhibition of Pim-2 reduces glycolysis, oxidative phosphorylation, and ATP production, thereby promoting apoptosis of malignant plasma cells [62]. In a recent review, Oudaert et al. (2022) highlighted the complex metabolic interplay between multiple myeloma cells and the bone marrow microenvironment, involving mechanisms such as mitochondrial transfer and the reprogramming of bioenergetic pathways including glycolysis, glutamine metabolism, and lipogenesis with emphasis on promising therapeutic targets such as *HK2*, *PFKFB*, and *PYCR1* [63].

The integration of multi-omics platforms including genomics, transcriptomics, proteomics, and metabolomics provides a comprehensive and dynamic view of the disease, enabling more refined risk stratification and the implementation of truly personalized therapies [40,64].

### 4.6. Radiomics

The application of radiomics to MM has emerged as a promising strategy to complement traditional imaging approaches and overcome the limitations inherent to bone marrow biopsy, which samples only a focal site and may fail to reflect the spatial heterogeneity of MM. Through modalities such as ^18^F-FDG PET/CT and whole-body MRI, radiomics extracts hundreds of quantitative features related to texture, shape, intensity, and tissue heterogeneity, converting medical images into measurable biomarkers. This capability is especially relevant in MM, a disease characterized by multiple skeletal and medullary involvement and marked biological heterogeneity, where variability between lesions may impact prognosis, therapeutic response, and risk of progression [65,66].

By comprehensively capturing the medullary and skeletal heterogeneity at a whole-body level, radiomics tools hold the potential to integrate MRD evaluation in a non-invasive and global manner, surpassing reliance on bone marrow biopsy from a single site. When combined with machine-learning models and possibly multi-omic or clinical data, radiomics signatures may, in the future, contribute to risk stratification, identify patients with deep and sustained responses, and anticipate relapse before being detectable by conventional molecular or cytometric methods. Although still under clinical validation, radiomics is shaping up as a key component of precision medicine in MM, with potential to inform individualized treatment decisions, optimize MRD monitoring, and improve long-term disease management [67,68].

### 4.7. Optical Genome Mapping (OGM)

Multiomics tools have brought drastic changes in the last decade, enabling precision medicine and promising results. Across all oncohematological diseases, a high rate of acquired balanced chromosomal translocations, which can lead to the emergence of fusion genes, is observed. These structural variants (SVs) are important drivers of neoplastic development and progression. Detection of VS in multiple myeloma remains challenging, as obtaining CD138+ cells is not always an easy task in routine laboratory practice. Therefore, a combination of techniques such as NGS, G-banded karyotyping, FISH, and CNV microarrays in a single sample is often necessary for detection [56,58,69].

OGM is an emerging molecular cytogenetic technology using fluorescently labeled high-molecular-weight genomic DNA capable of detecting SV and CNVs throughout the genome. It also features a simple workflow and straightforward bioinformatics pipelines, allowing analysis directly in the equipment’s software, without the need for a bioinformatician. Structural alterations such as deletions, translocations, and inversions can be detected as small as 500 bp, offering high resolution and coverage (up to 600× depending on the pipeline). Although we do not yet have multicenter studies demonstrating the clinical use of OGM in multiple myeloma, some findings have shown promise for the detection of SVs and CNVs in the genome of patients, especially when combined with NGS [39,69,70].

## 5. Perspectives

In the 21st century, despite significant advances in healthcare, a relatively common oncohematological disease remains largely unknown and incurable. Even within the medical community, there is a clear deficit in recognizing the initial clinical signs, and many patients are only diagnosed after sustaining bone fractures managed by orthopedic specialists. However, by the time this outcome occurs, numerous physicians may have already overlooked earlier diagnostic opportunities, often due to failing to request routine tests, such as serum protein electrophoresis and calcium levels [1].

To cure malignant diseases, early detection and intervention are crucial. MM itself is preceded by premalignant stages, and until recently, treatment was only recommended if the patient fulfilled the SLiM-CRAB criteria. Substantial debate persists among the medical community regarding earlier treatment, yet we can already note progress: patients with HR-SMM undergo therapy before symptomatic MM develops [20,21,34,71].

**Figure 2:**
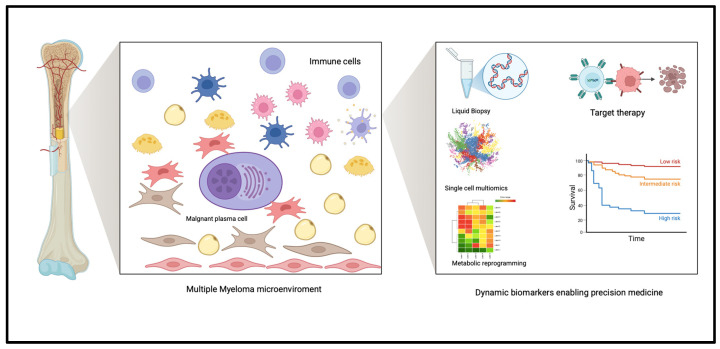
Bone marrow microenvironment–driven biomarkers enabling precision medicine in multiple myeloma. Malignant plasma cells interact with bone marrow stromal, immune, endothelial, and bone-resident cells, shaping tumor progression, immune dysfunction, and therapeutic resistance. These microenvironmental interactions generate dynamic, minimally invasive biomarkers, including liquid biopsy components, single-cell multiomics profiles, and metabolic signatures, enabling refined risk stratification and biologically guided therapeutic decision-making. Integrating these next-generation biomarkers supports precision medicine strategies beyond current guideline-based approaches. Graphical elements are illustrative and represent conceptual outputs of multiomic and biomarker-based analyses.

Current NCCN and IMWG guidelines rely primarily on cytogenetics, FLC ratio, and imaging for diagnosis and risk assessment. The biomarker classes discussed in this review, particularly liquid biopsy components and multiomic classifiers, offer a path toward next-generation precision medicine tools that may refine or even replace existing guideline-based algorithms. Their future integration could enable earlier detection, dynamic monitoring, and individualized therapeutic decision-making [1,20]. Figure 2 provides a conceptual overview of how microenvironment-derived biomarkers, including single-cell, metabolic, and liquid biopsy approaches, may inform risk stratification and precision-guided therapy.

## 6. Conclusions

MM is a highly complex and heterogeneous disease, and despite considerable advances, a definitive cure for this neoplasm has not yet been achieved. This new era of precision medicine offers renewed hope: the integration of emerging biomarkers with those already used for diagnosis, prognosis, and monitoring may enable more personalized, biologically informed, and potentially transformative therapies.

Although NCCN and IMWG guidelines continue to define the standard of care, emerging multiomic and liquid-biopsy-based biomarkers hold the potential to expand diagnostic precision and personalize therapy in ways that current algorithms cannot yet achieve. Bridging these innovations with guideline-based practice represents the next frontier in multiple myeloma management.

## Figures and Tables

**Table 1:** MGUS classification according to the Mayo Clinic risk model and recommended follow-up.

Classification	Diagnostic	Risk/Classification	Follow Up
Non-light chain MGUS (IgG/IgA/IgM)	-Serum MP < 3 g/dL -<10% clonal plasma cells in BM -No SLiM-CRAB features or amyloidosis	1. MP > 1.5 g/dL 2. Non-IgG isotype (IgA/IgM) 3. Abnormal FLC ratio Low risk: 0 factors Intermediate: 1–2 factors High risk: 3 factors	Initial tests: CBC, creatinine, calcium, SPEP/IFE, FLC Bone marrow biopsy: not routine; indicated only if M-protein ≥ 1.5 g/dL, abnormal FLC, unexplained anemia, renal impairment, hypercalcemia, bone lesions, or suspicion of amyloidosis IgM-specific: abdominal CT or ultrasound only if suspicion of LPL/WM Follow-up: • Low risk: repeat SPEP/FLC in 6 months → if stable, every 2–3 years • 1–2 risk factors: repeat in 6 months → then annually • High risk: every 3–6 months for 2 years → then annually Cardiac biomarkers (NT-proBNP, Troponin T): when symptoms or labs suggest amyloidosis, or in high-risk MGUS
Light-chain MGUS (LC-MGUS)	-Abnormal FLC ratio -No heavy-chain on IFE -Involved FLC < 150 mg/L 10% clonal plasma cells	Stratified separately by FLC ratio and involved chain level (Mayo LC-MGUS model); abnormal FLC ratio	Bone marrow biopsy: indicated only if unexplained anemia, renal failure, bone lesions, or suspicion of AL amyloidosis Follow-up: every 6 months for 2 years → then annually NT-proBNP/Troponin: mandatory if clinical suspicion of amyloidosis

Abbreviations: AL—Amyloid Light-chain; BM—Bone Marrow; CBC—Complete Blood Count; CT—computed tomography; CRAB—Hyper Calcemia, Renal failure, Anemia, Bone lesions; FLC—Free Light Chain; IFE—Immunofixation Electrophoresis; Ig—Immunoglobulin; LC-MGUS—Light-Chain Monoclonal Gammopathy of Undetermined Significance; LPL—Lymphoplasmacytic Lymphoma; MGUS—Monoclonal Gammopathy of Undetermined Significance; MP—M-protein; NT-proBNP—N-terminal pro–B-type Natriuretic Peptide; SLiM—≥60% plasma cells in BM; FLC ratio ≥100 with involved FLC ≥100 mg/L; >1 focal lesion on MRI (magnetic resonance imaging); SPEP—Serum Protein Electrophoresis; WM—Waldenstrom Macroglobulinemia.

**Table 2:** Drugs used to treat Multiple Myeloma.

Therapeutic Family	Drug Class	Mechanism of Action	Targets	Agents
Proteasome Pathway Targeting	Proteasome inhibitors	Blocks 26S proteasome → accumulation of misfolded proteins → apoptosis	PSMB5, ubiquitin–proteasome pathway	Bortezomib, Carfilzomib, Ixazomib
Immunomodulation	IMiDs	Immunomodulation, T/NK activation, anti-angiogenic	CRBN, Ikaros/Aiolos degradation	Thalidomide, Lenalidomide, Pomalidomide
	CELMoDs (next-gen IMiDs)	Potent cereblon E3 ligase modulators → enhanced Ikaros/Aiolos degradation	CRBN E3 ligase	Iberdomide, Mezigdomide
Monoclonal Antibody Therapy	Anti-CD38	ADCC/CDC; apoptosis	CD38	Daratumumab, Isatuximab
	Anti-SLAMF7	Immune activation (NK-mediated)	SLAMF7	Elotuzumab
T-cell-Redirecting Therapies	Bispecific antibodies (BsAbs)	Dual engagement of tumor Ag + CD3 → directs T-cell killing	BCMA × CD3, GPRC5D × CD3, FcRH5 × CD3	Teclistamab, Elranatamab, Talquetamab, Linvoseltamab
	CAR-T cell therapy	Autologous T cells engineered with CAR receptors	BCMA, GPRC5D	Ide-cel, Cilta-cel
Targeted Cytotoxic Delivery	Antibody–drug conjugates (ADCs)	mAb linked to toxic payload → internalized → cell death	BCMA	Belantamab mafodotin
DNA-Damage-Inducing Therapies	Alkylating agents	DNA crosslink → apoptosis	DNA (non-specific)	Melphalan, Cyclophosphamide
Corticosteroids	Glucocorticoids	Lympholytic, apoptotic → synergistic with all MM therapies	Glucocorticoid receptor	Dexamethasone, Prednisone
Bone Microenvironment Modulation	Bone-modifying agents	Inhibit osteoclast activation	RANK/RANKL pathway	Zoledronic acid, Pamidronate, Denosumab
Other Cytotoxic/Supportive Therapy	Traditional chemotherapy	DNA or microtubule disruption	Non-specific	Cisplatin, Doxorubicin, Vincristine, Etoposide

Abbreviations: ADCC—Antibody-Dependent Cellular Cytotoxicity; ADC—Antibody–Drug Conjugate; Ag—Antigen; BCMA—B-Cell Maturation Antigen; CAR-T—Chimeric Antigen Receptor T-cell; CDC—Complement-Dependent Cytotoxicity; CELMoD—Cereblon E3 Ligase Modulator (next-generation IMiDs); CRBN—Cereblon; DNA—Deoxyribonucleic Acid; FcRH5—Fc Receptor-Homolog 5; GPRC5D—G Protein-Coupled Receptor Class C Group 5 Member D; IMiD—Immunomodulatory Drug; mAb—Monoclonal Antibody; NK—Natural Killer; PSMB5—Proteasome Subunit Beta Type-5 (β5 subunit of 20S proteasome); RANKL—Receptor Activator of Nuclear Factor-κB Ligand; SLAMF7—Signaling Lymphocytic Activation Molecule Family Member 7.

## Data Availability

No new data were created or analyzed in this study.

## References

[1] Rajkumar S.V., Dimopoulos M.A., Palumbo A., Blade J., Merlini G., Mateos M.V., Kumar S., Hillengass J., Kastritis E., Richardson P. (2014). International Myeloma Working Group Updated Criteria for the Diagnosis of Multiple Myeloma. Lancet Oncol..

[2] Morgan G.J., Walker B.A., Davies F.E. (2012). The Genetic Architecture of Multiple Myeloma. Nat. Rev. Cancer.

[3] Wallington-Beddoe C.T., Mynott R.L. (2021). Prognostic and Predictive Biomarker Developments in Multiple Myeloma. J. Hematol. Oncol..

[4] Pinto V., Bergantim R., Caires H.R., Seca H., Guimarães J.E., Vasconcelos M.H. (2020). Multiple Myeloma: Available Therapies and Causes of Drug Resistance. Cancers.

[5] Agarwal A., Ghobrial I.M. (2013). Monoclonal Gammopathy of Undetermined Significance and Smoldering Multiple Myeloma: A Review of the Current Understanding of Epidemiology, Biology, Risk Stratification, and Management of Myeloma Precursor Disease. Clin. Cancer Res..

[6] Landgren O. (2021). Advances in MGUS Diagnosis, Risk Stratification, and Management: Introducing Myeloma-Defining Genomic Events. Hematology.

[7] Landgren O., Kyle R.A., Pfeiffer R.M., Katzmann J.A., Caporaso N.E., Hayes R.B., Dispenzieri A., Kumar S., Clark R.J., Baris D. (2009). Monoclonal Gammopathy of Undetermined Significance (MGUS) Consistently Precedes Multiple Myeloma: A Prospective Study. Blood.

[8] Ferreira B., Caetano J., Barahona F., Lopes R., Carneiro E., Costa-Silva B., João C. (2020). Liquid Biopsies for Multiple Myeloma in a Time of Precision Medicine. J. Mol. Med..

[9] Li S., Zhang E., Cai Z. (2023). Liquid Biopsy by Analysis of Circulating Myeloma Cells and Cell-Free Nucleic Acids: A Novel Noninvasive Approach of Disease Evaluation in Multiple Myeloma. Biomark. Res..

[10] Kyle R.A., Durie B.G.M., Rajkumar S.V., Landgren O., Blade J., Merlini G., Kröger N., Einsele H., Vesole D.H., Dimopoulos M. (2010). Monoclonal Gammopathy of Undetermined Significance (MGUS) and Smoldering (Asymptomatic) Multiple Myeloma: IMWG Consensus Perspectives Risk Factors for Progression and Guidelines for Monitoring and Management. Leukemia.

[11] Dispenzieri A., Katzmann J.A., Kyle R.A., Larson D.R., Melton L.J., Colby C.L., Therneau T.M., Clark R., Kumar S.K., Bradwell A. (2010). Prevalence and Risk of Progression of Light-Chain Monoclonal Gammopathy of Undetermined Significance: A Retrospective Population-Based Cohort Study. Lancet.

[12] Kyle R.A., Larson D.R., Therneau T.M., Dispenzieri A., Kumar S., Cerhan J.R., Rajkumar S.V. (2018). Long-Term Follow-up of Monoclonal Gammopathy of Undetermined Significance. N. Engl. J. Med..

[13] Rajkumar S.V., Kyle R.A., Therneau T.M., Melton L.J., Bradwell A.R., Clark R.J., Larson D.R., Plevak M.F., Dispenzieri A., Katzmann J.A. (2005). Serum Free Light Chain Ratio Is an Independent Risk Factor for Progression in Monoclonal Gammopathy of Undetermined Significance. Blood.

[14] Kyle R.A., Therneau T.M., Rajkumar S.V., Offord J.R., Larson D.R., Plevak M.F., Melton L.J. (2002). A Long-Term Study of Prognosis in Monoclonal Gammopathy of Undetermined Significance. N. Engl. J. Med..

[15] Dispenzieri A., Kyle R.A., Katzmann J.A., Therneau T.M., Larson D., Benson J., Clark R.J., Melton L.J., Gertz M.A., Kumar S.K. (2008). Immunoglobulin Free Light Chain Ratio Is an Independent Risk Factor for Progression of Smoldering (Asymptomatic) Multiple Myeloma. Blood.

[16] Mateos M.V., Kumar S., Dimopoulos M.A., González-Calle V., Kastritis E., Hajek R., De Larrea C.F., Morgan G.J., Merlini G., Goldschmidt H. (2020). International Myeloma Working Group Risk Stratification Model for Smoldering Multiple Myeloma (SMM). Blood Cancer J..

[17] Landgren O., Hofmann J.N., McShane C.M., Santo L., Hultcrantz M., Korde N., Mailankody S., Kazandjian D., Murata K., Thoren K. (2019). Association of Immune Marker Changes with Progression of Monoclonal Gammopathy of Undetermined Significance to Multiple Myeloma. JAMA Oncol..

[18] Kyle R.A., Remstein E.D., Therneau T.M., Dispenzieri A., Kurtin P.J., Hodnefield J.M., Larson D.R., Plevak M.F., Jelinek D.F., Fonseca R. (2007). Clinical Course and Prognosis of Smoldering (Asymptomatic) Multiple Myeloma. N. Engl. J. Med..

[19] Rajkumar S.V. (2012). Preventive Strategies in Monoclonal Gammopathy of Undetermined Significance and Smoldering Multiple Myeloma. Am. J. Hematol..

[20] Kumar S.K., Anderson L.D., Baljevic M., Baz R., Campagnaro E., Costello C., Derman B., Devarakonda S., Elsedawy N., Godara N. (2025). NCCN Guidelines Version 4.2026.

[21] Kumar S.K., Anderson L.D., Baljevic M., Baz R., Campagnaro E., Costello C., Derman B., Devarakonda S., Elsedawy N., Godara N. (2025). NCCN Guidelines Version 2.2026.

[22] Visram A., Rajkumar S.V., Kapoor P., Dispenzieri A., Lacy M.Q., Gertz M.A., Buadi F.K., Hayman S.R., Dingli D., Kourelis T. (2022). Correction: Monoclonal Proteinuria Predicts Progression Risk in Asymptomatic Multiple Myeloma with a Free Light Chain Ratio ≥100. Leukemia.

[23] Zhou L., Yu Q., Wei G., Wang L., Huang Y., Hu K., Hu Y., Huang H. (2021). Measuring the Global, Regional, and National Burden of Multiple Myeloma from 1990 to 2019. BMC Cancer.

[24] Misund K., Keane N., Stein C.K., Asmann Y.W., Day G., Welsh S., Van Wier S.A., Riggs D.L., Ahmann G., Chesi M. (2020). MYC Dysregulation in the Progression of Multiple Myeloma. Leukemia.

[25] Campo E., Jaffe E.S., Cook J.R., Quintanilla-Martinez L., Swerdlow S.H., Anderson K.C., Brousset P., Cerroni L., de Leval L., Dirnhofer S. (2022). The International Consensus Classification of Mature Lymphoid Neoplasms: A Report from the Clinical Advisory Committee. Blood.

[26] Fend F., Dogan A., Cook J.R. (2023). Plasma Cell Neoplasms and Related Entities Evolution in Diagnosis and Classification. Virchows Arch..

[27] Greipp P.R., Miguel J.S., Dune B.G.M., Crowley J.J., Barlogie B., Bladé J., Boccadoro M., Child J.A., Harousseau J.L., Kyle R.A. (2005). International Staging System for Multiple Myeloma. J. Clin. Oncol..

[28] Palumbo A., Avet-Loiseau H., Oliva S., Lokhorst H.M., Goldschmidt H., Rosinol L., Richardson P., Caltagirone S., Lahuerta J.J., Facon T. (2015). Revised International Staging System for Multiple Myeloma: A Report from International Myeloma Working Group. J. Clin. Oncol..

[29] Chawla S.S., Kumar S.K., Dispenzieri A., Greenberg A.J., Larson D.R., Kyle R.A., Lacy M.Q., Gertz M.A., Rajkumar S.V. (2015). Clinical Course and Prognosis of Non-Secretory Multiple Myeloma. Eur. J. Haematol..

[30] Kyle R.A., Gertz M.A., Witzig T.E., Lust J.A., Lacy M.Q., Dispenzieri A., Fonseca R., Rajkumar S.V., Offord J.R., Larson D.R. (2003). Review of 1027 Patients with Newly Diagnosed Multiple Myeloma. Mayo Clin. Proc..

[31] Gertz M.A., Dingli D. (2014). How We Manage Autologous Stem Cell Transplantation for Patients with Multiple Myeloma. Blood.

[32] Charliński G., Vesole D.H., Jurczyszyn A. (2021). Rapid Progress in the Use of Immunomodulatory Drugs and Cereblon E3 Ligase Modulators in the Treatment of Multiple Myeloma. Cancers.

[33] Berdeja J.G., Madduri D., Usmani S.Z., Jakubowiak A., Agha M., Cohen A.D., Stewart A.K., Hari P., Htut M., Lesokhin A. (2021). Ciltacabtagene Autoleucel, a B-Cell Maturation Antigen-Directed Chimeric Antigen Receptor T-Cell Therapy in Patients with Relapsed or Refractory Multiple Myeloma (CARTITUDE-1): A Phase 1b/2 Open-Label Study. Lancet.

[34] Lipof J.J., Pan D., Kumar A.D., Chung A., Chari A. (2025). AQUILA: Dousing the Embers of Smoldering Multiple Myeloma with Daratumumab Monotherapy. Hematol..

[35] Bolli N., Avet-Loiseau H., Wedge D.C., Van Loo P., Alexandrov L.B., Martincorena I., Dawson K.J., Iorio F., Nik-Zainal S., Bignell G.R. (2014). Heterogeneity of Genomic Evolution and Mutational Profiles in Multiple Myeloma. Nat. Commun..

[36] Walker B.A., Wardell C.P., Melchor L., Brioli A., Johnson D.C., Kaiser M.F., Mirabella F., Lopez-Corral L., Humphray S., Murray L. (2014). Intraclonal Heterogeneity Is a Critical Early Event in the Development of Myeloma and Precedes the Development of Clinical Symptoms. Leukemia.

[37] Sonneveld P., Avet-Loiseau H., Lonial S., Usmani S., Siegel D., Anderson K.C., Chng W.J., Moreau P., Attal M., Kyle R.A. (2016). Treatment of Multiple Myeloma with High-Risk Cytogenetics: A Consensus of the International Myeloma Working Group. Blood.

[38] Le G.N., Bones J., Coyne M., Bazou D., Dowling P., O’Gorman P., Larkin A.M. (2019). Current and Future Biomarkers for Risk-Stratification and Treatment Personalisation in Multiple Myeloma. Mol. Omics.

[39] Giguère A., Raymond-Bouchard I., Collin V., Claveau J.S., Hébert J., LeBlanc R. (2023). Optical Genome Mapping Reveals the Complex Genetic Landscape of Myeloma. Cancers.

[40] Malyutina A., Sergeev P., Huber J., Miettinen J.J., Bolomsky A., Bao J., Parsons A.O., Muller A., Marella N., van Duin M. (2024). Multi-Omics Data Integration Reveals Molecular Mechanisms of Carfilzomib Resistance in Multiple Myeloma. bioRxiv.

[41] Allegra A., Cicero N., Tonacci A., Musolino C., Gangemi S. (2022). Circular RNA as a Novel Biomarker for Diagnosis and Prognosis and Potential Therapeutic Targets in Multiple Myeloma. Cancers.

[42] Reale A., Khong T., Mithraprabhu S., Spencer A. (2021). Translational Potential of RNA Derived from Extracellular Vesicles in Multiple Myeloma. Front. Oncol..

[43] Nilsson R.J.A., Balaj L., Hulleman E., Van Rijn S., Pegtel D.M., Walraven M., Widmark A., Gerritsen W.R., Verheul H.M., Vandertop W.P. (2011). Blood Platelets Contain Tumor-Derived RNA Biomarkers. Blood.

[44] Kis O., Kaedbey R., Chow S., Danesh A., Dowar M., Li T., Li Z., Liu J., Mansour M., Masih-Khan E. (2017). Circulating Tumour DNA Sequence Analysis as an Alternative to Multiple Myeloma Bone Marrow Aspirates. Nat. Commun..

[45] Paiva B., Paino T., Sayagues J.M., Garayoa M., San-Segundo L., Martín M., Mota I., Sanchez M.L., Bárcena P., Aires-Mejia I. (2013). Detailed Characterization of Multiple Myeloma Circulating Tumor Cells Shows Unique Phenotypic, Cytogenetic, Functional, and Circadian Distribution Profile. Blood.

[46] Brennecke J., Stark A., Russell R.B., Cohen S.M. (2005). Principles of MicroRNA-Target Recognition. PLoS Biol..

[47] Reinhart B.J., Slack F.J., Basson M., Pasquienelll A.E., Bettlnger J.C., Rougvle A.E., Horvitz H.R., Ruvkun G. (2000). The 21-Nucleotide Let-7 RNA Regulates Developmental Timing in Caenorhabditis Elegans. Nature.

[48] Al Masri A., Price-Troska T., Chesi M., Chung T.-H., Kim S., Carpten J., Bergsagel P.L., Fonseca R. (2005). MicroRNA Expression Analysis in Multiple Myeloma. Blood.

[49] Kubiczkova L., Kryukov F., Slaby O., Dementyeva E., Jarkovsky J., Nekvindova J., Radova L., Greslikova H., Kuglik P., Vetesnikova E. (2014). Circulating Serum MicroRNAs as Novel Diagnostic and Prognostic Biomarkers for Multiple Myeloma and Monoclonal Gammopathy of Undetermined Significance. Haematologica.

[50] Zhang Z.Y., Li Y.C., Geng C.Y., Wang H.J., Chen W.M. (2019). Potential Relationship between Clinical Significance and Serum Exosomal MiRNAs in Patients with Multiple Myeloma. Biomed. Res. Int..

[51] Leslie M. (2010). Beyond Clotting: The Powers of Platelets. Science (1979).

[52] Sudha P., Ahsan A., Ashby C., Kausar T., Khera A., Kazeroun M.H., Hsu C.C., Wang L., Fitzsimons E., Salminen O. (2022). Myeloma Genome Project Panel Is a Comprehensive Targeted Genomics Panel for Molecular Profiling of Patients with Multiple Myeloma. Clin. Cancer Res..

[53] Ren Y., Liu M., Fang J., Wang L., Yu L., Xue Y., Zhao W., Liu J., Jin Y., Tian Y. (2024). NGS-Based Immunoglobulin Gene Sequencing for Diagnosis and MRD Monitoring of Multiple Myeloma. Blood.

[54] Bolli N., Biancon G., Moarii M., Gimondi S., Li Y., de Philippis C., Maura F., Sathiaseelan V., Tai Y.T., Mudie L. (2018). Analysis of the Genomic Landscape of Multiple Myeloma Highlights Novel Prognostic Markers and Disease Subgroups. Leukemia.

[55] Rosa-Rosa J.M., Cuenca I., Medina A., Vázquez I., Sánchez-delaCruz A., Buenache N., Sánchez R., Jiménez C., Rosiñol L., Gutiérrez N.C. (2022). NGS-Based Molecular Karyotyping of Multiple Myeloma: Results from the GEM12 Clinical Trial. Cancers.

[56] Walker B.A. (2018). Whole Exome Sequencing in Multiple Myeloma to Identify Somatic Single Nucleotide Variants and Key Translocations Involving Immunoglobulin Loci and MYC. Methods in Molecular Biology.

[57] Manier S., Park J., Capelletti M., Bustoros M., Freeman S.S., Ha G., Rhoades J., Liu C.J., Huynh D., Reed S.C. (2018). Whole-Exome Sequencing of Cell-Free DNA and Circulating Tumor Cells in Multiple Myeloma. Nat. Commun..

[58] Kumar A., Adhikari S., Kankainen M., Heckman C.A. (2021). Comparison of Structural and Short Variants Detected by Linked-read and Whole-exome Sequencing in Multiple Myeloma. Cancers.

[59] Alaterre E., Vikova V., Kassambara A., Bruyer A., Robert N., Requirand G., Bret C., Herbaux C., Vincent L., Cartron G. (2021). Rna-Sequencing-Based Transcriptomic Score with Prognostic and Theranostic Values in Multiple Myeloma. J. Pers. Med..

[60] Yu Z., Qiu B., Li L., Xu J., Zhou H., Niu T. (2022). An Emerging Prognosis Prediction Model for Multiple Myeloma: Hypoxia-Immune Related Microenvironmental Gene Signature. Front. Oncol..

[61] Yue L., Zeng P., Li Y., Chai Y., Wu C., Gao B. (2022). Nontargeted and Targeted Metabolomics Approaches Reveal the Key Amino Acid Alterations Involved in Multiple Myeloma. PeerJ.

[62] Liu Z., Guo Y., Liu X., Cao P., Liu H., Dong X., Ding K., Fu R. (2023). Pim-2 Kinase Regulates Energy Metabolism in Multiple Myeloma. Cancers.

[63] Oudaert I., Van der Vreken A., Maes A., De Bruyne E., De Veirman K., Vanderkerken K., Menu E. (2022). Metabolic Cross-Talk within the Bone Marrow Milieu: Focus on Multiple Myeloma. Exp. Hematol. Oncol..

[64] Huber J., Malyutina A., Bolomsky A., Zojer N., Schreder M., Schneller A., Pfeiffer C., Miettinen J., Tang J., Heckman C. (2022). P-104: Multi-Omics Data Integration Reveals Molecular Targets of Carfilzomib Resistance in Multiple Myeloma. Clin. Lymphoma Myeloma Leuk..

[65] Milara E., Alonso R., Masseing L., Seiffert A.P., Gómez-Grande A., Gómez E.J., Martínez-López J., Sánchez-González P. (2023). Radiomics Analysis of Bone Marrow Biopsy Locations in [18F] FDG PET/CT Images for Measurable Residual Disease Assessment in Multiple Myeloma. Phys. Eng. Sci. Med..

[66] Zhong H., Huang D., Wu J., Chen X., Chen Y., Huang C. (2023). 18F-FDG PET/CT Based Radiomics Features Improve Prediction of Prognosis: Multiple Machine Learning Algorithms and Multimodality Applications for Multiple Myeloma. BMC Med. Imaging.

[67] Filippi L., Ferrari C., Nuvoli S., Bianconi F., Donner D., Marongiu A., Mammucci P., Vultaggio V., Chierichetti F., Rubini G. (2024). Pet-Radiomics in Lymphoma and Multiple Myeloma: Update of Current Literature. Clin. Transl. Imaging.

[68] Klontzas M.E., Triantafyllou M., Leventis D., Koltsakis E., Kalarakis G., Tzortzakakis A., Karantanas A.H. (2023). Radiomics Analysis for Multiple Myeloma: A Systematic Review with Radiomics Quality Scoring. Diagnostics.

[69] Kriegova E., Fillerova R., Minarik J., Savara J., Manakova J., Petrackova A., Dihel M., Balcarkova J., Krhovska P., Pika T. (2021). Whole-Genome Optical Mapping of Bone-Marrow Myeloma Cells Reveals Association of Extramedullary Multiple Myeloma with Chromosome 1 Abnormalities. Sci. Rep..

[70] Toruner G.A., Hu S., Loghavi S., OK C.Y., Tang Z., Wei Q., Kanagal-Shamanna R., Medeiros L.J., Tang G. (2025). Clinical Utility of Optical Genome Mapping as an Additional Tool in a Standard Cytogenetic Workup in Hematological Malignancies. Cancers.

[71] Joao C., Bergantim R., Santos J., Afonso C., Bernardo P., Coelho H., Costa C., Esteves G., Freitas J.G., Gerivaz R. (2023). Multiple Myeloma Treatment Guidelines by the Portuguese Group of Multiple Myeloma. Acta Med. Port..

